# Diabetes is associated with increased risk of death in COVID‐19 hospitalizations in Mexico 2020: A retrospective cohort study

**DOI:** 10.1002/hsr2.1416

**Published:** 2023-07-05

**Authors:** Alexander A. Huang, Samuel Y. Huang

**Affiliations:** ^1^ Department of MD Education Northwestern University Feinberg School of Medicine Chicago Illinois USA; ^2^ Department of Internal Medicine Virginia Commonwealth University School of Medicine Richmond Virginia USA

**Keywords:** COVID‐19, diabetes, epidemiology, global health, Mexico, survival analysis

## Abstract

**Background and Aim:**

The COVID‐19 disease course can be thought of as a function of prior risk factors consisting of comorbidities and outcomes. Survival analysis data for diabetic patients with COVID‐19 from an up to date and representative sample can increase efficiency in resource allocation. The study aimed to quantify mortality in Mexico for individuals with diabetes in the setting of COVID‐19 hospitalization.

**Methods:**

This retrospective cohort study utilized publicly available data from the Mexican Federal Government, covering the period from April 14, 2020, to December 20, 2020 (last accessed). Survival analysis techniques were applied, including Kaplan–Meier curves to estimate survival probabilities, log‐rank tests to compare survival between groups, Cox proportional hazard models to assess the association between diabetes and mortality risk, and restricted mean survival time (RMST) analyses to measure the average survival time.

**Results:**

A total of 402,388 adults age greater than 18 with COVID‐19 were used in the analysis. Mean age = 16.16 (SD = 15.55), 214,161 males (53%). Twenty‐day Kaplan–Meier estimates of mortality were 32% for COVID‐19 patients with diabetes and 10.2% for those without diabetes with log‐rank *p* < 0.01. Univariable analysis showed increased mortality in diabetic patients (hazard ratio [HR]: 3.61, 95% confidence interval [CI]: 3.54–3.67, *p* < 0.01) showing a 254% increase in death. After controlling for confounding variables, multivariate analysis continued to show increased mortality in diabetics (HR: 1.37, 95% CI: 1.29–1.44, *p* < 0.01) indicating a 37% increase in death. Multivariable RMST at Day 20 showed in Mexico, hospitalized COVID‐19 patients were associated with less mean survival time by 2.01 days (*p* < 0.01) and a 10% increased mortality (*p* < 0.01).

**Conclusions:**

In the present analysis, COVID‐19 patients with diabetes in Mexico had shorter survival times. Further interventions aimed at improving comorbidities in the population, particularly in individuals with diabetes, may contribute to better outcomes in COVID‐19 patients.

## 1 INTRODUCTION

COVID‐19 is caused by SARS‐CoV‐2 virus first identified in Wuhan, China around December 2019 and spread globally.^1^, ^2^ The virus spread through internationally and reached Mexico February 28, 2020.^2^, ^3^ Since then, over 7 million confirmed cases of COVID‐19 and over 300,000 have occurred in Mexico.^4^, ^5^ A total of 200,000,000 doses have been administered, which roughly approximates to three‐quarters of the population receiving a dose.^6^, ^7^ Even so, Latin America continues to have a disproportionate burden of disease.^8^, ^9^

Mexico has increased comorbidities and age. These comorbidities are associated with increased risk of death and worse outcomes.^10^, ^11^ Mexico has the highest overweight and obesity rates with a prevalence of diabetes of over 10% roughly equating to 13 million Mexicans.^12^ This number has increased since 2006 by 7% and continues to grow.^13^ Diabetes plays a role in increasing risk of contraction and the outcome of many upper respiratory infections including the common cold and influenza.^14^, ^15^ The proposed mechanism is in disrupting the body's ability to mount a response and immune dysregulation. Increased inflammation increases the assault to the alveoli.^16^ Diabetes also does not exist in isolation but is comorbid with a lot of other risk factors that are not independently associated such as hypertension and death.

Numerous reports from China, Italy, and other countries have consistently demonstrated that patients with diabetes are more susceptible to severe forms of COVID‐19.^17^, ^18^ Studies have shown that individuals with diabetes and COVID‐19 pneumonia are more likely to require intensive care, experience organ failure, and exhibit hypercoagulability.^18^, ^19^, ^20^, ^21^, ^22^ The mortality rates among COVID‐19 patients with diabetes have been alarmingly high, ranging from 11% to 33% in different populations.^23^ A retrospective study conducted in China revealed that patients with pre‐existing type 2 diabetes required more medical interventions and presented with multiple organ failure compared to nondiabetic individuals.^24^ These findings were supported by observations from the French COVID CORONADO cohort, where diabetic patients had a higher risk of death within 28 days after hospitalization.^17^ While the existing body of research consistently associates diabetes with severe COVID‐19 outcomes, certain gaps and limitations in previous studies need to be addressed. Many studies have not adequately accounted for confounding factors such as the type of diabetes, disease duration, glycemic control, presence of diabetic complications, and comorbidities like obesity, hypertension, and cardiovascular diseases.^25^ These factors contribute to the heterogeneity of the diabetic population and may impact the association between diabetes and COVID‐19 morbidity. Moreover, studies conducted in different countries have reported varying mortality rates among COVID‐19 patients with diabetes. This highlights the need for comprehensive investigations that consider the diversity of populations, healthcare systems, and socioeconomic factors.^26^ Additionally, there is a lack of research that explores the impact of interventions targeting comorbidities, particularly in individuals with diabetes, on the outcomes of COVID‐19 patients.

To address these gaps and limitations, the present study focuses on the Mexican population and seeks to investigate the relationship between diabetes and COVID‐19 mortality. Patients that contract COVID‐19 have a range of symptoms ranging from asymptomatic to death.^27^ Previous studies have looked at mortality in COVID‐19, but have not looked at the specific subset of individuals in Mexico with comorbid diabetes with the level of statistical analyses utilizing both Cox proportional hazards and restricted mean survival time.^11^, ^28^, ^29^ Proper delineation of the effect size of diabetes can help improve clinical reasoning from physicians and streamline resource allocation. The findings of this study are expected to contribute to a more thorough understanding of the impact of diabetes on COVID‐19 outcomes in the Mexican population. The results may help inform resource allocation and guide interventions aimed at improving comorbidities, particularly in individuals with diabetes, to achieve better outcomes for COVID‐19 patients. This study seeks to fill the gap in large‐scale Mexican population specific analysis of mortality risk from diabetes in COVID‐19 hospitalized patients.

## 2 METHODS

This study was a retrospective study that used data from the Mexican Federal Government. The data set was published and validated by the Epidemiological Surveillance System for Viral Respiratory Diseases of the Mexican Ministry of Health and was approved by the ethics committees of the Ministry of Health.

### 2.1 Data set and cohort selection

The COVID‐19 Mexican Patients Data set was used to examine the characteristics, demographics, and outcomes of the Mexican population during the COVID‐19 pandemic. The data set was collected by the Mexican Federal Government and released by the Mexican Ministry of Health. It includes data from 475 Viral Respiratory Disease Monitoring Units and medical units and contains information on hospitalized COVID‐positive patients from April 14, 2020 to December 20, 2020. A total of 402,388 patients with complete death data were included in the present analysis.

### 2.2 Determination of COVID‐19

COVID‐19 diagnosis was determined by testing for the presence of the SARS‐CoV‐2 antigen using nasal swabs. The test was conducted at various monitoring and medical units by the Mexican Government, and results were available in a timely manner. A positive COVID‐19 status was defined as the presence of the SARS‐CoV‐2 antigen, while a negative status was defined as the absence of the antigen.

### 2.3 Variables

This study aims to investigate the impact of diabetes on the prognosis of COVID‐19 in Mexican patients on the probability of death in the hospital among diabetic patients. This is a large, national, retrospective cohort study that will provide valuable insights into the relationship between diabetes and COVID‐19 outcomes, which can inform the development of effective mitigation strategies and improve patient triaging. Information regarding individuals' sex, age, country of origin, existing health conditions (such as hypertension, diabetes, and obesity), smoking habits, and pregnancy status were documented for each person. The COVID‐19 status includes recorded data on antigen test outcomes and whether an antigen sample was collected. Throughout their stay at medical facilities, no details concerning the patients' progress were made accessible to the public.

### 2.4 Statistical analysis

The demographic and diabetes‐related characteristics of patients with a positive SARS‐CoV‐2 antigen were analyzed using descriptive statistics. *T*‐tests and *χ* ^2^ tests were used to compare the patients in relation to relevant covariates. The primary endpoint of the study was survival, which was defined as the time from symptom onset to death and censored at the last enrollment date for adult hospitalized COVID‐19 patients. Kaplan–Meier curves were plotted to estimate the survival curve, and a log‐rank test was used to determine the statistical significance of the survival times between adult hospitalized patients with and without diabetes. A Cox proportional hazards model was used to calculate the hazard ratio and 95% confidence interval (CI) for the treatment effect. All statistical tests were two‐sided and a *p*‐value of less than 0.05 was considered statistically significant. In addition to overall survival, the restricted means survival time (RMST) was also calculated for diabetic and nondiabetic hospitalized COVID‐19 adult patients after propensity matching to control for confounders. This method fitted a parametric survival model to the data to estimate the mean survival time for the two groups.^30^

## 3 RESULTS

Table 1 presents the demographic variables and key covariates of COVID‐19‐positive patients in the Mexican Patients Data set released by the Mexican Ministry of Health. A total of 402,388 adults who were hospitalized with COVID‐19 and had complete death data were included in the study. Of these, 53% were male and 47% were female, with a mean age of 46.16 (SD = 15.55). Common comorbidities among these patients included hypertension (20%), diabetes (16%), and obesity (19%), with 7% of the individuals being smokers.

**Table 1:** Patient demographics and covariates.

	All individuals	Diabetic	Nondiabetic	
Total Individuals	402,388	66,082	336,306	
Male	214,161 (53%)	35,486 (54%)	178,675 (53%)	*p* = 0.01
Female	188,227 (47%)	30,596 (46%)	157,631 (47%)	*p* = 0.01
Age	46.16 ± 15.55	57.74 ± 13.05	43.89 ± 14.98	*p* < 0.01
Native	385,671 (96%)	63,284 (96%)	322,387 (96%)	*p* < 0.01
Diabetes	66,082 (16%)	66,082 (100%)	0 (0%)	*p* < 0.01
Hypertension	81,604 (20%)	35,450 (54%)	46,154 (14%)	*p* < 0.01
Obesity	77,003 (19%)	18,454 (28%)	58,549 (17%)	*p* < 0.01
Smoking	29,996 (7%)	5,276 (8%)	24,720 (7%)	*p* < 0.01
Pneumonia	88,064 (22%)	28,105 (43%)	59,959 (18%)	*p* < 0.01
ICU	10,285 (3%)	3,509 (5%)	6,776 (2%)	*p* = 0.01
Intubation	23,670 (6%)	8,433 (13%)	15,237 (5%)	*p* < 0.01
Death	55,356 (14%)	21,129 (32%)	34,227 (10%)	*p* < 0.01

**Figure 1:**
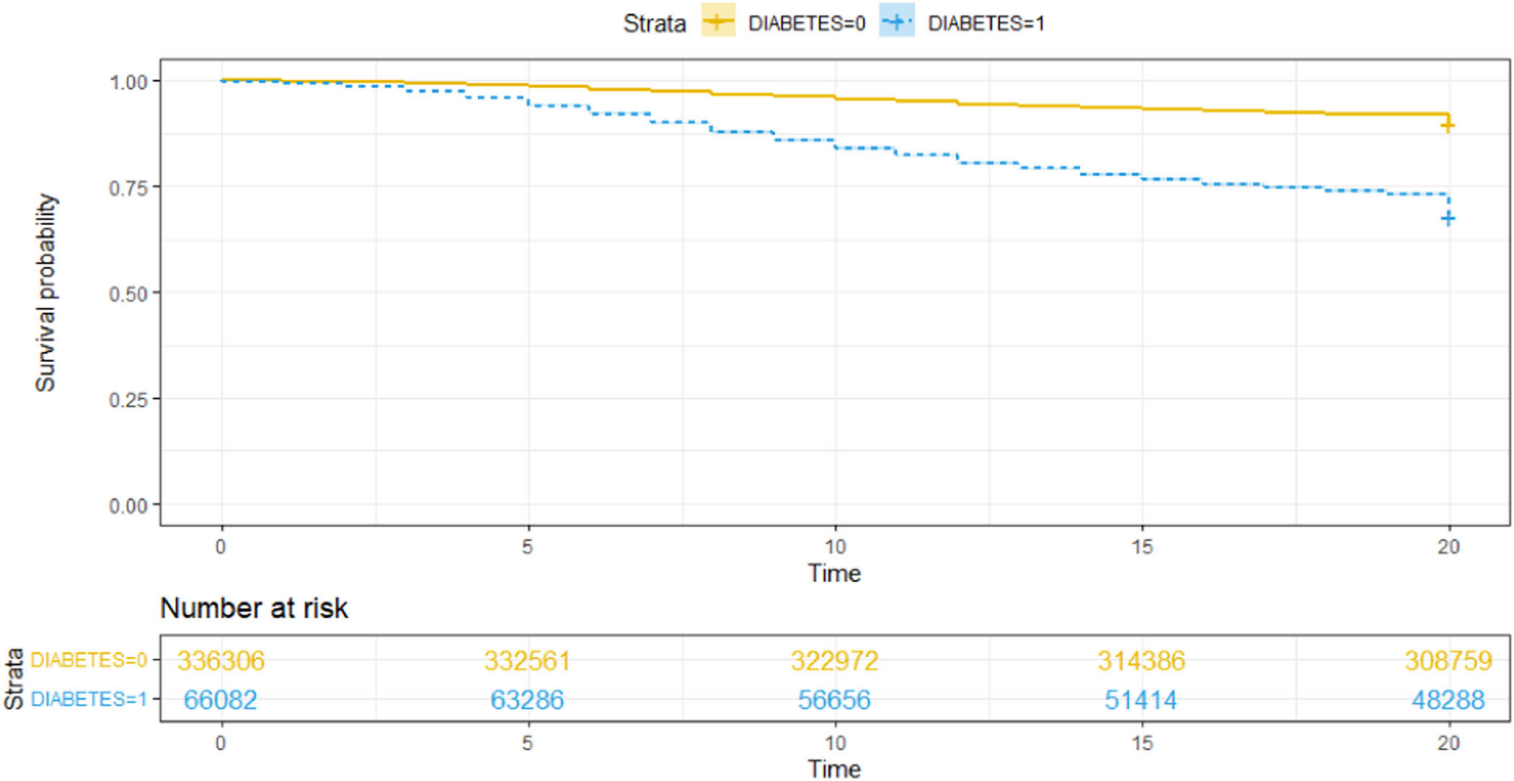
Multivariable Kaplan–Meier survival plots comparing diabetic and nondiabetic patients.

Figure 1 displays Kaplan Meier survival plots comparing the survival of diabetic and nondiabetic patients. The survival analysis includes 402,388 individuals, of which 55,356 died. A log‐rank test revealed that there was a statistically significant difference in survival between hospitalized COVID‐19 patients with and without diabetes (*p* < 0.01). The graph also satisfies the proportional hazard assumption as the curves do not intersect during the analyzed period. Tables 2a and 2b present the results of the univariable and multivariable Cox proportional hazards models, which are both significant for diabetes. In the univariable model, diabetic patients had a 261% increased risk of mortality (*p* < 0.01) at any time during the analyzed period. This increased risk persisted even after adjusting for confounders, with diabetic patients having a 37% increased risk of mortality (*p* < 0.01).

**Table 2a:** Univariable Cox proportional survival model.

	HR	95% CI	*p* Value
Diabetes	3.61	(3.54–3.67)	*p* < 0.001

**Table 2b:** Multivariable Cox proportional survival model.

	HR	95% CI	*p* Value
Diabetes	1.37	(1.29–1.44)	*p* < 0.01
Male	1.5	(1.44–1.57)	*p* < 0.01
Native	1.23	(0.89–1.57)	*p* = 0.22
Hypertension	1.62	(1.54–1.69)	*p* < 0.01
Obesity	1.02	(0.92–1.12)	*p* = 0.71
Smoking	1.13	(1–1.25)	*p* = 0.06

**Figure 2:**
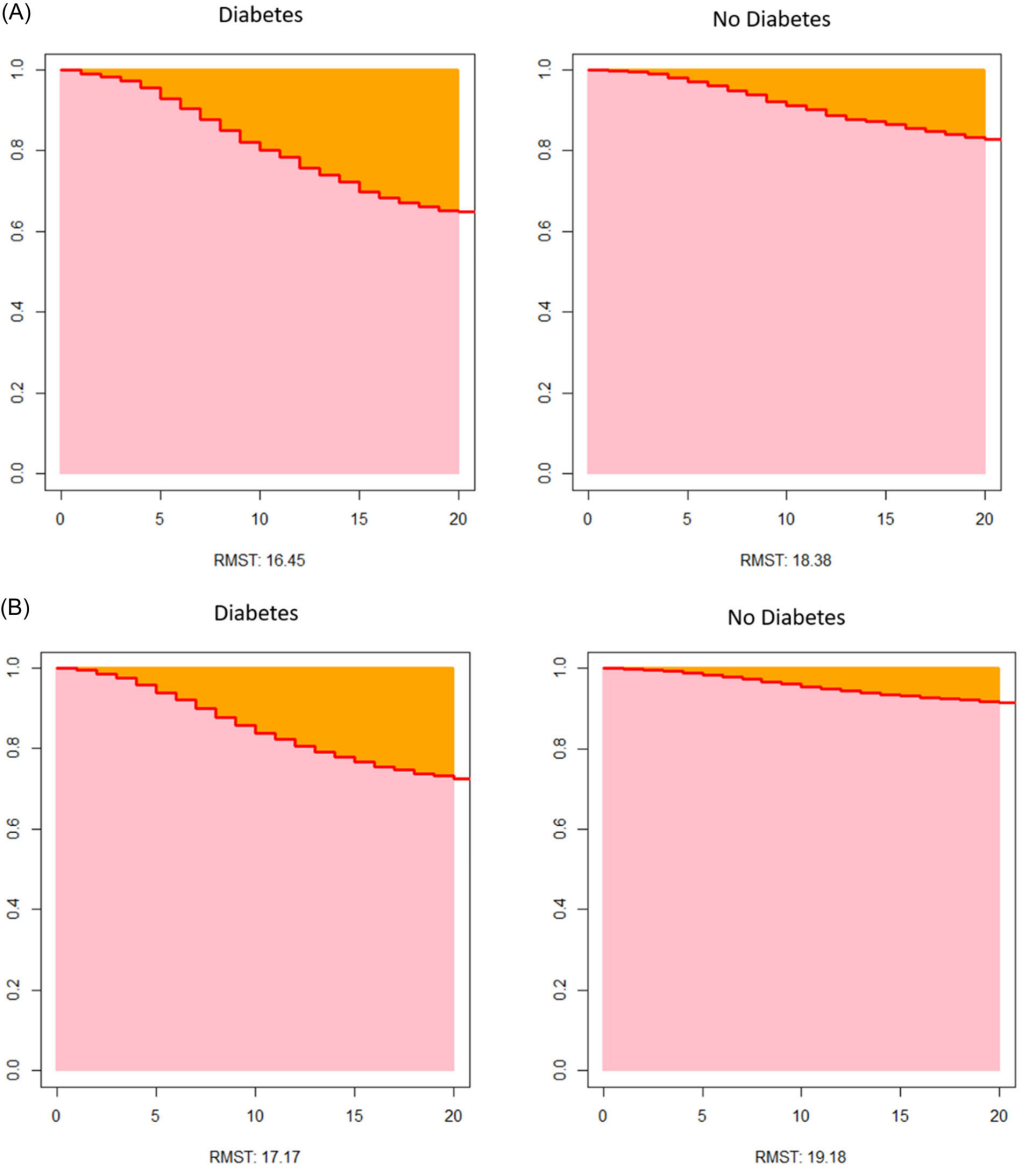
(A) RMST univariable model. (B) RMST multivariable model. RMST, restricted mean survival time.

Tables 3a and 3b provide information on the RMST. In the univariable RMST model, diabetic patients had a decreased survival time of 2.01 days (*p* < 0.01) and a 10% increased mortality (*p* < 0.01) compared to nondiabetic patients. The above can be visually shown in Figure 2A and b with the RMST graphs comparing survival times between diabetic and nondiabetic patients who have contracted COVID‐19. The association between diabetes and mortality persisted in the multivariable model controlling for confounders, with diabetic patients having a decreased survival time of 1.93 days (*p* < 0.01) and a 10% increased mortality (*p* < 0.01) compared to nondiabetic patients.

**Table 3a:** RMST univariable.

	RMST estimate	95% CI	*p* Value
RMST (Diabetes) − (No diabetes)	−2.01	(−2.05 to −1.97)	*p* < 0.01
RMST (Diabetes)/(No diabetes)	0.90	(0.89–0.90)	*p* < 0.01

Abbreviation: RMST, restricted mean survival time.

**Table 3b:** RMST multivariable.

	RMST estimate	95% CI	*p* Value
RMST (Diabetes) – (No diabetes)	−1.93	(−2.25 to −1.61)	*p* < 0.01
RMST (Diabetes)/(No diabetes)	0.90	(0.88–0.91)	*p* < 0.01

Abbreviation: RMST, restricted mean survival time.

## 4 DISCUSSION

In this large, retrospective cohort study of the Mexican population, adults with readily available information on death outcomes and a positive SARS‐CoV‐2 antigen test who also had comorbid diabetes had a statistically significant increased risk of death from COVID‐19. The Mexican cohort in this study had a high prevalence of comorbidities, including hypertension (20%), diabetes (16%), and obesity (19%), among patients with COVID‐19. These findings are consistent with previous research on adults in the United States, which has shown that individuals with diabetes are at an increased risk of severe illness and complications from COVID‐19.^31^

The results demonstrate demographic numbers that are consistent with rates reported in other studies such as the 21.6% prevalence among hospitalized patients with COVID‐19 in Wuhan.^32^ The data is also representative of the greater than 10% prevalence in Mexico for the general population.^13^ Studies have suggested that individuals with diabetes may be at an increased risk for poor outcomes in COVID‐19.^19^ The rate of death in hospitalized patients in the present study (32%) is similar to mortality rates reported in other countries such as Italy (25.2%) and Belgium (29.9%).^20^, ^21^ Research has indicated that people with diabetes may be more likely to experience severe illness and complications from COVID‐19.^22^ Diabetes and high blood sugar alone has been found to result in poorer outcomes.^33^ Many descriptive studies have found high rates of COVID‐19 and diabetes concurrently.^34^, ^35^

The results of the present survival analysis is consistent with other survival studies find survival analysis studies. According to a study enrolling patients for a 3‐month period in 2020 at Dr. Soetomo General Hospital in Indonesia there was a decrease in probability of survival in patients with a metric that included a decrease in white cell count, diabetes, older age.^36^ A National Cohort Study in England with ICU patients found 23% increase in mortality among diabetic patients (hazard ratio [HR]: 1.23, 95% CI: 1.14–1.42, *p* < 0.01) in 2020.^29^ A 2021 survival analysis in Uganda found a 22.5% mortality in COVID‐19 patients hospitalized and 40% higher hazard ratios (HR: 1.4, CI: 1–2.2, *p* < 0.01).^28^ In different regions of Brazil in 2020 they found a roughly 10% (*p* < 0.01) increased prevalence of diabetes among patients with COVID‐19 compared to those suggesting diabetes could both increase risk of getting COVID‐19 and the outcome associated.^37^

There are studies that suggest there can be a bitemporal association between diabetes and COVID‐19. A case report found newly diagnosed diabetes in a recent COVID‐19 patient.^38^ A meta‐analysis done by Zhang et al. found 1.17 (relative risk [RR]: 1.17, 1.02–1.34) RR of developing diabetes after COVID‐19.^39^

The proposed mechanism of diabetes stems beyond just that of COVID‐19, but also includes worsening outcomes and increased contraction of upper respiratory infections (URIs).^14^, ^15^ Diabetes impairs the body's ability to amount an immune response from constant and chronic inflammation and damage to blood vessels.^40^, ^41^, ^42^ Diabetes also disrupts the bodies immune regulation.^43^, ^44^ Diabetes occurs alongside other comorbidities that each also individually contribute to worser outcomes. Tailored interventions, such as targeted education on diabetes management, close monitoring of glycemic control, and early identification of complications, could potentially mitigate the increased mortality risk in this vulnerable population.

One of the major advantages of the present study was the use of a large sample size, which enabled us to detect significant differences between groups with greater precision. The sample was validated by the Mexican Ministry of Health and was representative of the adult population in Mexico due to the inclusion of data from 475 monitoring units across the country. We also controlled for confounding variables in the present analysis to accurately evaluate the relationship between diabetes and COVID‐19 outcomes. These features contributed to the credibility and accuracy of this study's results.

There are a few points to keep in mind when interpreting the findings of the present study. As an observational and retrospective study, it is not possible to determine a cause‐and‐effect relationship between the variables being examined.^45^ Additionally, the lack of temporal data on diabetes makes it challenging to understand the impact of COVID‐19 on the diabetic status of patients over time. While the sample size of the study is large, the data were collected from the population of Mexico, and the results may not be applicable to other populations, particularly those outside of Mexico. As a study that is reliant on reported data from individuals, there could be reporting bias. It is important to consider these limitations when interpreting the results of the study.

## 5 CONCLUSIONS

This analysis supports previous research demonstrating that comorbidities, particularly diabetes, increase the risk of mortality in patients with COVID‐19 in Mexico and around the world. Further research is necessary to fully understand the relationship between COVID‐19 and diabetes and to improve outcomes in this population. Further implementation of interventions targeting the improvement of comorbidities, specifically in individuals with diabetes, has the potential to enhance outcomes in individuals with COVID‐19.

## AUTHOR CONTRIBUTIONS

**Alexander A. Huang**: Conceptualization; supervision; writing—original draft; writing—review and editing. **Samuel Y. Huang**: Conceptualization; data curation; formal analysis; investigation; methodology; project administration; resources; software; supervision; validation; visualization; writing—original draft; writing—review and editing.

## CONFLICT OF INTEREST STATEMENT

The authors declare no conflict of interest.

## ETHICAL APPROVAL

The methods behind acquisition and analysis of the data are described by the Epidemiological Surveillance System for Viral Respiratory Diseases of the Mexican Ministry of Health.

## TRANSPARENCY STATEMENT

The lead author Samuel Y. Huang affirms that this manuscript is an honest, accurate, and transparent account of the study being reported; that no important aspects of the study have been omitted; and that any discrepancies from the study as planned (and, if relevant, registered) have been explained.

## Data Availability

The data from this cohort can be found on COVID‐19 database on the Mexican Federal Government website. Data described in the manuscript is publicly and freely available without restriction at https://datos.gob.mx/
